# Host and immunosuppression-related factors influencing fibrosis occurrence post liver transplantation

**DOI:** 10.3389/fphar.2022.1042664

**Published:** 2022-10-18

**Authors:** Speranta Iacob, Razvan Iacob, Ioana Manea, Mihaela Uta, Andrei Chiosa, Mona Dumbrava, Gabriel Becheanu, Luminita Stoica, Codruta Popa, Vlad Brasoveanu, Doina Hrehoret, Cristian Gheorghe, Liana Gheorghe, Simona Dima, Irinel Popescu

**Affiliations:** ^1^ Gastroenterology Department, University of Medicine and Pharmacy “Carol Davila”, Bucharest, Romania; ^2^ Center for Excellence in Translational Medicine, Bucharest, Romania; ^3^ Fundeni Clinical Institute, Bucharest, Romania

**Keywords:** graft fibrosis, tacrolimus, mycophenolate mofetil, CYP3A5 genotype, apoptosis, hepatocytes culture, liver tranpslant, TBX21

## Abstract

Post liver transplantation (LT) fibrosis has a negative impact on graft function. Cytokine production in the host immune response after LT may contribute to the variable CYP3A-dependent immunosuppressive drug disposition, with subsequent impact on liver fibrogenesis, together with host-related factors. We aimed to investigate whether the cytochrome P4503A5*3 (CYP3A5*3) or TBX21 genotypes impact post-LT liver fibrogenesis. Furthermore, the impact of immunosuppressants on cellular apoptosis has been evaluated using human hepatocytes harvested from cirrhotic explanted livers. We have enrolled 98 LT recipients that were followed for occurrence of liver fibrosis for at least 12 months. There was a statistically significant higher trough level of TAC in patients with homozygous CC-TBX21 genotype (7.83 ± 2.84 ng/ml) vs. 5.66 ± 2.16 ng/ml in patients without this genotype (*p* = 0.009). The following variables were identified as risk factors for fibrosis ≥2: donor age (*p* = 0.02), neutrophil to lymphocyte ratio (*p* = 0.04) and TBX21 genotype CC (*p* = 0.009). In the cell culture model cytometry analysis has indicated the lowest apoptotic cells percentage in human cirrhotic hepatocytes cultures treated with mycophenolate mofetil (MMF) (5%) and TAC + MMF (2%) whereas the highest apoptosis percentage was registered for the TAC alone (11%). The gene expression results are concordant to cytometry study results, indicating the lowest apoptotic effect for MMF and MMF + TAC immunosuppressive regimens. The allele 1993C of the SNP rs4794067 may predispose to the development of late significant fibrosis of the liver graft. MMF-based regimens have a favourable anti-apoptotic profile *in vitro*, supporting its use in case of LT recipients at high risk for liver graft fibrosis.

## 1 Introduction

The availability of the calcineurin inhibitors (CNIs) cyclosporine (CsA) (Calne et al., 1978) and tacrolimus (TAC) (Starzl et al., 1989) has completely changed the prognosis of transplanted patients. Currently, more than 90% of all patients receiving a graft are treated post-transplant with CNIs. The combination of a CNI and mycophenolate mofetil (MMF) or sirolimus (SRL) are commonly used for maintenance immunosuppression following liver transplantation (LT) (Iacob et al., 2009). However, there are post-LT complications immunosuppression-related that negatively impact survival and quality of life. *In vitro* and *in vivo* studies have demonstrated profibrogenic properties of CNIs with increased hepatic and renal collagen deposition triggered by a high expression of transforming growth factor beta 1 and extracellular matrix genes (Frizell et al., 1994). Newer studies suggest a role of these agents in promoting and enhancing cell-death in non-liver cell types like pancreatic cells, renal and prostate cells, thereby driving an inflammatory response that promotes fibrosis (Khanna et al., 1997; Giordano et al., 2006; Choi et al., 2008; Bouvier et al., 2009). On the other hand, CNIs at therapeutic concentrations did not affect murine hepatocyte apoptosis, but combination with MMF significantly enhanced cell death. By contrast, SRL/MMF combination did not significantly reduce hepatocyte viability or promote apoptosis (Nguyen et al., 2009). However, hepatic stellate cells (HSCs) play the critical role in liver fibrosis; reversal of liver fibrosis can be achieved through the apoptosis of activated HSCs. Immunosuppressive agents may also affect the life cycle of HSCs. One study (Kabat-Koperska et al., 2016) showed that the treatment with MMF induced human HSC apoptosis and reduced collagen alpha 1 expression compared to CsA or SRL treatment. Thus, some immunosuppressive agents may have antifibrotic properties and not profibrotic.

As previously mentioned, the immunosuppressive regimen with TAC is essential for patients after LT. The cytochrome P450 3A (CYP3A) subfamily and P-glycoprotein in human liver and intestine can contribute to inter and intra-individual differences in the pharmacokinetics of TAC (Thervet et al., 2003; Rojas et al., 2015). A single nucleotide polymorphism (SNP) in the CYP3A5 gene involving an A to G transition at position 6986 within intron 3 was found strongly associated with CYP3A5 protein expression. At least one CYP3A5*1 allele were found to express large amounts of CYP3A5 protein, whereas homozygous for the CYP3A5*3 allele did not express significant quantities of CYP3A5 protein, which results in a truncated protein and a severe decrease of functional CYP3A5 (Hesselink et al., 2014). The CYP3A5*1 SNP is currently the most promising biomarker for tailoring TAC treatment.

There is accumulating evidence that regulatory T cells (Tregs) have a crucial role in immune tolerance and long-term graft survival. T-box21 gene (transcription factor T-bet – T –box expressed in T cells - TBX21), as well as GATA 3 (GATA binding protein 3) and FOXP3 (forkhead box P3) genes constitute the principle regulators for the differentiation of Th1, Th2 and Tregs (Szabo et al., 2000; Evans and Jenner, 2013). These T cells are implicated in different post-transplant complications and are influenced by the immunosuppressive drugs. TBX21 is a key transcriptional activator of Th1 cell differentiation. T-bet plays an essential role in Th1/Th2 balance, where it is the master regulator of Th1 cell fate through promotion of Th1 cytokines and inhibition of Th2 cytokines (Szabo et al., 2000; Usui et al., 2006).

The SNP rs4794067 represents a T to C substitution at position -1993 in the promoter region of the TBX21 gene. The -1993C allele of the SNP rs4794067 is associated with a reduced promoter activity, when compared with the -1993T allele. The expression level of TBX21 is lower in individuals with the -1993C allele than in individuals with the -1993T allele (Suttner et al., 2009; Li et al., 2011) and hence there is a decreased proinflammatory cytokines generated by Th2 or Th17 cells.

The main objective of the present paper was to investigate whether the cytochrome P450 3A5*3 (CYP3A5*3) or TBX21 genotypes affect TAC pharmacokinetics and to evaluate their potential impact on liver fibrogenesis post-LT, after controlling for the host-related factors. Furthermore, the impact of immunosuppressants on cellular apoptosis has been evaluated using human hepatocytes harvested from cirrhotic explanted livers.

## 2 Materials and methods

### 2.1 Clinical study

Between October 2018 and March 2020, we have enrolled 98 LT recipients that were followed for occurrence of liver fibrosis for at least 12 months. Non-invasive evaluation of the liver was performed at the moment of inclusion into the study (Fibroscan^®^ with CAP and FIB4) for detection of fibrosis stage ≥2 and/or steatosis grade 3 occurrence. All clinical data were collected in a database. Buffy coat from patients were obtained for genotyping of CYP3A5*3 (rs776746) and TBX21 (rs4794067) polymorphisms by Taqman SNP Genotyping Assays (Thermo Scientific). All patients signed an informed consent; the study was approved by the Ethics Committee of Fundeni Clinical Institute (126769/29.09.2018) and conducted in accordance with guidelines for human studies.

### 2.2 Statistical analysis

Quantitative variables were expressed as mean ± standard deviation or median and interquartile range. Categorical variables were expressed as frequencies and percentages. Quantitative variables were compared by T-Student test or Mann Whitney U test. Pearson correlation coefficient was used to correlate quantitative variables. Qualitative variables were compared by Chi-squared or Fisher exact test. Cox regression analysis was performed to identify predictors of the outcome and log-rank test was used for comparison of Kaplan Meyer curves. Results with *p* ≤ 0.05 were considered statistically significant.

### 2.3 Cell culture models

In this study human hepatocytes were isolated from cirrhotic explanted livers. All livers were processed within a few hours after harvesting. They were transferred to the hepatocyte isolation lab on ice, in University of Wisconsin solution under sterile conditions. Human hepatocyte isolation was performed using collagenase perfusion with the technique described by Seglen et al. (Seglen, 1976; Mitry et al., 2002).

Cirrhotic hepatocytes have been cultured in low glucose DMEM culture medium and have been treated for 24 h with different concentrations and of immunosupresants ranging from 10 nM to 100 µM: TAC, SRL, MMF, or combinations (TAC + SRL, MMF + TAC). At 24 h apoptosis and necrosis was assessed using Tali™ Apoptosis Kit - Annexin V Alexa Fluor™ 488 & Propidium Iodide (Thermo Scintific). Gene expression has been assessed by qRT-PCR using a microarray of 19 genes significant for apoptosis. Relative expression of selected genes was evaluated by the Real-Time PCR System using SYBR Green Master Mix (Thermo Fisher Scientific Waltham, MA, United States), with beta-actin as a reference gene. Cirrhotic liver hepatocytes cultures, untreated with immunosupresants were used as a reference for gene expression quantification. The studied genes and the primers used for specific amplification of the mRNA are depicted in Supplementary Table S1.

Cirrhotic human hepatocytes differ from hepatocytes isolated from normal liver by a rapid *in vitro* conversion to a mesenchymal proliferative phenotype that offers advantages as a study *in vitro* model due to the cellular expansion capacity permitting experimental repeatability.

During our study there were used 3 different cellular cultures isolated from explanted liver during LT procedures. During the same procedure were isolated hepatocytes and also non-parenchymal hepatic cells by centrifugation technique on Percoll gradient of the supernatant obtained at the first wash of the cellular suspension resulting from the enzymatic digestion of the hepatic parenchyma by collagenase solution.

## 3 Results

### 3.1 Study of the impact of the CYP3A5 and TBX21 genotypes following LT

#### 3.1.1 General characteristics of the LT patients

There were included 98 patients: 56.1% men and 43.9% women, median age at inclusion 59 years and median time since LT was 62.6 months; the median age of the donors (91 cadaveric and 7 living) was 42 years; gender of the donors were: 42.3% women and 57.7% men. The median time of warm ischemia was 39 min and the median cold ischemia time was 4.75 h.

Etiology of liver cirrhosis was: hepatitis C (HCV) in 59.2%, hepatitis B and delta coinfection in 19.4%, alcohol related cirrhosis in 7.1%, autoimmune liver disease in 8.1% and others in 6.2% of cases. Hepatocellular carcinoma (HCC) was present in 30.6% of patients. All patients transplanted for HCV had cured hepatitis C after LT with the new direct antivirals. Diabetes mellitus was encountered in 20.4% of patients after LT. 73.5% of patients received TAC, 7.1% CsA and 19.4% SRL. MMF was associated in 28.6% of patients. A significantly higher proportion of patients with HCC received SRL (33.3% vs. 8.8%, *p* = 0.002). Table 1 shows the biochemical parameters studied in our cohort. In Table 2 are shown the differences between LT recipients with and without HCV-related cirrhosis.

**TABLE 1:** Biochemical characteristics and noninvasive liver fibrosis assessment of the analysed cohort.

Variable	Median value (range)
ALT (U/L)	27.5 (9.5–183)
AST (U/L)	24 (12.2–101)
GGT (U/L)	23 (6–887)
Alkaline phosphatase (U/L)	92 (25.2–417)
Total bilirubin (mg/dl)	0.69 (0.2–4.84)
Creatinine (mg/dl)	1.08 (0.56–2.36)
Uric acid (mg/dl)	5.4 (2.7–12.4)
Serum albumine (mg/dl)	4.56 (2.9–5.62)
Glycemia (mg/dl)	108.6 (72.1–209)
Total Cholesterol (mg/dl)	189 (40–331)
LDL cholesterol (mg/dl)	115.6 (51.4–261)
HDL cholesterol (mg/dl)	50.1 (27–91)
Triglycerides (mg/dl)	106 (40–306)
NLR (neutrophil to lymphocyte ratio)	2.42 (0.78–12.16)
PLR (platelet to lymphocyte ratio)	119.5 (42.3–304.7)
FIB-4	1.51 (0.57–11.58)
Liver stiffness measurement by Fibroscan (kPa)	5.5 (2.8–48)
CAP (dB/m)	274.5 (100–400)

**TABLE 2:** Analysis of the variables in patients with cured recurrent C hepatitis after LT vs. patients transplanted for other causes of cirrhosis.

Variable	HCV (+) group (n = 58)	HCV (-) group (n = 40)	p value
**Age (years)**	59.8 ± 7.8	52.6 ± 11.8	**0.0005**
**FIB-4**	2.41 ± 0.3	1.65 ± 0.2	**0.04**
Liver stiffness (kPa)	8.08 ± 0.8	7.31 ± 1.3	0.56
CAP (dB/m)	273.2 ± 65.0	252.1 ± 62.5	0.11
NLR (neutrophil to lymphocyte ratio)	2.79 ± 0.2	2.72 ± 0.2	0.83
PLR (platelet to lymphocyte ratio)	122.8 ± 46.2	127.3 ± 51.2	0.65
ALT (UI/L)	35.1 ± 3.8	34.6 ± 4.8	0.92
AST (UI/L)	30.4 ± 2.5	26.5 ± 2.2	0.28
GGT (UI/L)	54.2 ± 16.2	69.8 ± 23.1	0.57
Diabetes mellitus type II	25.9%	12.5%	0.10
**TBX21_CC genotype**	8.6%	27.5%	**0.01**
TBX21_CT genotype	43.1%	27.5%	0.11
TBX21_TT genotype	47.4%	45%	0.81
CYP3A5_CT genotype	13.8%	22.5%	0.26
CYP3A5_CC genotype	91.5%	82.1%	0.23

There was a moderate correlation between FIB-4 values and liver stiffness measured by Fibroscan^®^ (r = 0.51, *p* < 0.0001).

#### 3.1.2 Analysis of studied variables according to the CYP3A5 or TBX21 genes

In the studied cohort the frequency of CYP3A5-CC genotype was 88% and of CT genotype 12%; whereas the distribution of the TBX21 genotypes was as follows: CC genotype 17.5%; CT genotype 36.1% and TT genotype 46.4%.

Aminotransferases and gamma glutamyl transpeptidase did not differ at 7 and 14 days following LT according to the CYP3A5 or TBX21 genotypes. However, total bilirubin value was significantly higher at Day 7 and 14 post-LT in patients with CYP3A5-CC vs. CT genotype; creatinine values at 1 year after LT were significantly higher in patients with CYP3A5 CT vs. CC genotypes (1.22 ± 0.34 mg/dl vs. 1.05 ± 0.24 mg/dl, *p* = 0.01) where as glomerular filtration rates (GFR) were significantly lower in CT vs. CC genotypes (*p* = 0.04). TBX21 genotype did not influence the total bilirubin value early after LT or the creatinine/GFR values 1 year after LT.

There was no statistically significant difference between dose used or trough levels of TAC/SRL or CsA in patients with CC vs. CT CYP3A5 genotypes. On the other hand, TBX21 CC genotype was associated with a higher trough levels of TAC compared to other genotypes (*p* = 0.009), but not with a higher dose of TAC taken by the patient.

There was also no statistical difference between patients with CYP3A5 - CC genotype vs. CT with regard to FIB-4 values (*p* = 0.26), liver stiffness (*p* = 0.51) or CAP (*p* = 0.15) obtained by Fibroscan^®^. Patients with genotype TT of TBX21 gene had a statistically significantly higher steatosis grade evaluated by CAP compared to other genotypes (*p* = 0.009), but fibrosis stage evaluated by FIB-4 or liver stiffness did not variate according to genotype.

Inflammatory markers such as PLR or NLR did not differ according to CYP3A5 or TBX21 genotypes.

#### 3.1.3 Results of the cox regression analysis

The following variables were included in the univariate Cox regression analysis having as outcome occurrence of significant fibrosis stage (≥2) after LT (fibrosis evaluated by both concordant Fibroscan >8.1 kPa and FIB-4 >1.45): genotype CC of CYP3A5 gene; genotypes TT and CC of TBX21 gene; age of recipient and of the donor; presence of post-LT diabetes mellitus; HCV etiology of liver cirrhosis at LT; use of TAC, SRL and MMF; NLR and PLR values as well as steatosis grade evaluated by CAP.

Risk factors for fibrosis stage ≥2 were: TBX21 CC genotype (HR = 2.53, *p* = 0.009), NLR value (HR = 1.2, *p* = 0.04) and older donor age (HR = 1.1, *p* = 0.02). Independent risk factors for significant fibrosis stage after LT were only TBX21 CC genotype (*p* = 0.0001) and increased donor age (*p* = 0.0008).

Patients with TBX21 gene CC allele had a significantly lower graft survival rate compared to patients with CT/TT genotypes of TBX21 gene (median survival 51.4 vs. 102.6 months, *p* = 0.007). Patients receiving a combination of low dose TAC (aiming a trough level of 2–4 ng/ml) and MMF had a significantly lower percentage of significant graft fibrosis compared to patients with TAC monotherapy (27.4% vs. 47.1%, *p* = 0.03).

### 3.2 Study of the apoptosis process in liver cell culture models

#### 3.2.1 Apoptosis induced by the immunosuppression medication in the hepatocytes cultures

Apoptosis process induced by IS was studied in hepatocytes isolated from cirrhotic livers after treatment with TAC, SRL, MMF and combinations (TAC + SRL; TAC + MMF) according to the current standards of therapy of the LT recipients. There were studied the following increasing IS concentrations in order to identify an apoptotic effect different from the toxic-necrotic effect (10nM, 100nM, 1 μM, 10 μM, 100 μM).

**FIGURE 1:**
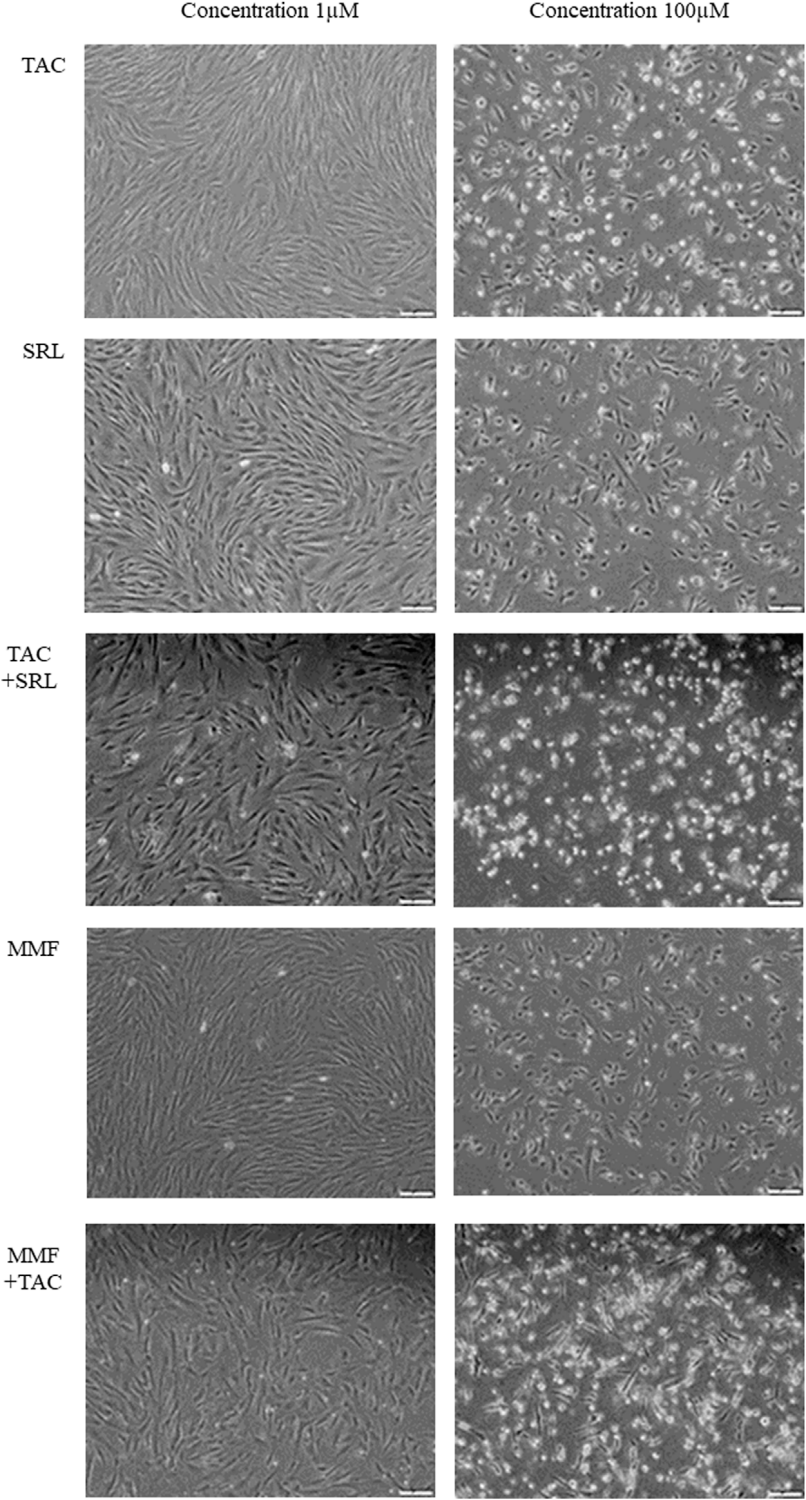
Examination of the hepatocyte cultures isolated from human cirrhotic liver after treatment with immunosuppression medication at a concentration of 1 µM and respectively 100 µM (Inverted microscope, phase contrast, 40X).

For the concentration of 10 nM of the IS the maximum percentage of apoptotic and necrotic cells for the combination of TAC + SRL was 19% and a low level of apoptosis of only 2% was noted for MMF or TAC + MMF. At a concentration of 1 µM the cumulated maximum apoptotic and necrotic cell percentage was 17% for TAC + SRL and only 3–5% of apoptotic cells for MMF or TAC + MMF. At a high concentration of 100 µM of IS, the level of apoptosis for TAC and MMF was still reduced, but for SRL and the combination of TAC and MMF there were registered high levels of cellular toxicity manifested by both apoptosis and cellular necrosis. Association of TAC + SRL at this concentration led to apoptosis and massive cellular necrosis with marked alteration of the cellular viability. According to the data obtained in the study of the cellular apoptosis induced by the IS on the adherent cultures of hepatocytes isolated from the human cirrhosic liver, we established as a study model for other cellular types the concentration of 1 µM where the apoptotic effect is evident, but without any significant toxic effect, manifested by the rate of cellular proliferation and cell death (Figure 1).

#### 3.2.2 Culture of non-parenchymal hepatic cells

For liver non-parenchymal cells, the level of apoptosis induced by the IS at a concentration of 1 µM is similar to that registered for the hepatocytes isolated from the cirrhotic liver, of approximate 5–7% of the cellular population without significant variations according to the type of IS. The most pronounced toxic effect is registered for the association of TAC + SRL and is significantly higher compared to the hepatocytes and is attributed to the cell necrosis.

#### 3.2.3 3D Co-culture of hepatocytes and non-parenchymal liver cells

In the co-culture systems the level of cellular apoptosis is low between 4 and 6%, even at high doses of immunosuppression.

### 3.3 Quantification of the gene expression for the significant genes for the cellular apoptosis

#### 3.3.1 In human hepatocyte cell cultures isolated from cirrhotic liver

**FIGURE 2:**
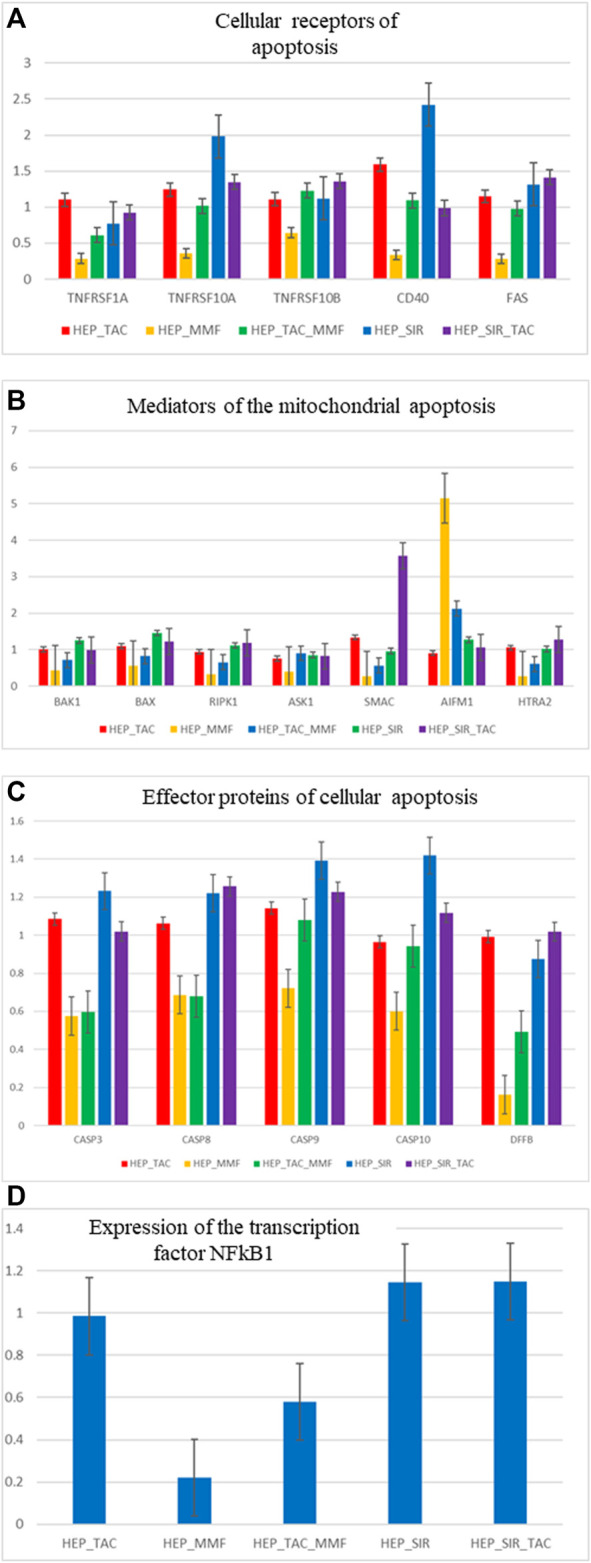
**(A**,**B**,**C**,**D)** Relative gene expression quantification (fold change) for significant genes involved in the process of cellular apoptosis, in hepatocyte cultures isolated from the cirrhotic liver after treatment with immunosuppressive drugs (1 µM).

The following genes were analysed according to their function: cellular receptors mediating a cellular apoptosis (TNFRSF1A-TNFR1, TNFTRS10A-TRAILR1, TNFRSF10B-TRAILR2, CD40 and FAS), genes specific to mithocondrial apoptosis (BAK1, BAX, RIPK1, ASK1-MAP3K5, SMAC-DIABLO, AIFM1, HTRA2) and effector proteins of apoptosis (CASP3, CASP8, CASP9, CASP10, DFFB-CAD), as well as the transcription factor significant for the cellular apoptosis NFkB1. In Figure 2 are presented the results of the relative quantification of the gene expression for the significant genes implicated in the cellular apoptosis in the hepatocyte cultures isolated from the cirrhotic liver following treatment with immunosuppressive drugs. Gene expression analysis indicated that MMF induces an antiapoptotic effect mediated by the decrease of the membrane receptors expression of the apoptosis, decrease of the gene expression that mediates cellular apoptosis, decrease of the genes that mediates mithocondrial apoptosis (excepting AIFM1), decrease of the effector proteins receptors of the apoptosis especially DFFB-CAD and a significant decrease of the transcription factor NFkB1. These results are concordant with the effects demonstrated in the cytometry studies of the cell cultures that indicated the lowest level of cellular apoptosis after treatment of the hepatocytes with MMF and MMF + TAC.

*In the non-parenchymal hepatic cell cultures* there is an unchanged expression for the studied genes with the exception of the overexpression of the SMAC gene in cell cultures treated with TAC. Results are also concordant to the cytometry studies.

Quantification of the apoptosis gene expression in the *3D co-culture of hepatocytes and non-parenchymal liver cells* revealed a favourable profile of the markers of cellular apoptosis in case of the MMF and TAC combination. For this association there is a significant underexpression of the TRAILR1, BAK, BAX and SMAC-DIABLO genes, specific markers of mitochondrial apoptosis and underexpression of the caspases 8, 9 and 10. There is also an overexpression of RIPK1, specific marker for necroptosis in the 3D cultures treated with TAC. There is also an overexpression of AIFM1 concordant with the results registered for the 2D hepatocyte cultures.

## 4 Discussions

LT is an established therapy associated with an excellent improvement in patients’ life expectancy over 70% at 10 years (Jadlowiec and Taner, 2016). However, an accelerated course of hepatic fibrosis may occur in LT patients despite normal or slightly abnormal liver blood tests (Gelson et al., 2010; Feng et al., 2018.), with concomitant allograft dysfunction. This late graft dysfunction is usually multifactorial (Kok et al., 2019; Iacob et al., 2021). Tailoring the immunosuppressive regimen has been proposed as a strategy to regulate fibrogenesis in the post-transplant period. Numerous intrinsic and extrinsic parameters influence TAC pharmacokinetic parameters, and variations in the expression and/or activity of drug metabolizing enzymes and transporters, in general supported by single nucleotide polimorphisms (SNPs), received much attention in the last years (Sakaeda, 2004). Of particular importance is the impact of variants in the genes encoding P450 cytochromes 3A (CYP3A4 and 3A5), which control TAC hepatic metabolism and intestinal absorption (Uesugi et al., 2014). However, in our paper this SNP had no influence on TAC trough levels and fibrosis occurrence after LT.

On the other hand, profile and Th1/Th2 ratio plays a strong role in fibrosis occurrence and can be affected by the activation of the hepatic stellate cells (Xu et al., 2012). Co-culture of the hepatic stellate cells with T CD4^+^ lymphocytes lead to inhibition of the Th1 cells response and increase of the Th2 response. In the presence of the SNP rs4794067, representing a T to C substitution at position -1993 in the promoter region of the TBX21 gene, associated with a reduced promoter activity and a lower TBX21 (Suttner et al., 2009; Li et al., 2011), as well as a shift of the Th1/Th2 ratio towards Th2, activation of these cells leading to a more pronounced fibrosis in this subgroup of patients. In addition, there is activation of the Th17 cells with eliberation of proinflammatory citokines. Induction of Th17 cells, defined as CD4^+^ or CD8^+^ IL17 producing cells, is highly dependent of the signals of citokines produced by other T cells populations or dendritic cells (Tesmer et al., 2008). Th17 cell response is propagated by the IL23 and IL21 citokines and IL17 production is antagonized by T-bet transcription factor and by the IFN gamma, IL4 and IL2 citokines (Bettelli et al., 2008). Thus, in the case of TBX21 -1993C allele of the SNP rs4794067 there is association with the predominance of the Th17 cell activity. TBX21 SNP rs4794067 was already associated with reumathoid artritis, asthma or type 1 diabetes mellitus (Sasaki et al., 2004; Chae et al., 2009), but no association was found with acute cellular LT rejection (Thude et al., 2019).

The role of TBX21 as a determinant signal for maturation of NK cells in late stages and their effector function is largely accepted nowadays. Recent studies have shown that non-parenchymal liver cells have important function in the tolerance induction following LT (Knechtle and Kwun., 2009). Kupffer cells proved to have a dual performance in the pathological changes that occur after LT, Th1 cells correlated with rejection and Th2 predominance favoured graft acceptance and immunological tolerance (Li et al., 2022).

Programmed cellular death occurs through different pathways including autophagy, necroptosis and apoptosis. There are lots of data that establish hepatocyte apoptosis as a leading force for fibrogenesis in various causes of hepatocyte injury (Guicciardi and Gores, 2010). Apoptotic hepatocytes are eliminated by Kupffer and hepatic stellate cells. Elimination of the apoptotic bodies by hepatic stellate cells results in their activation and TGF-beta secretion, a key factor in stimulation of liver fibrosis. Kupffer cells with phagocytated apoptotic hepatocytes secrete TGF-beta leading to a pro-apoptotic response through stellate cells activation. There was demonstrated that patients with LT had significantly higher apoptotic markers compared to subjects with normal liver (Shojaie et al., 2020). This fact is linked to the immunosuppressor agents used following transplantation that have cytotoxic and pro-apoptotic effects. Studies of the CNIs on cellular death are conflictual. Cyclosporine proved to increase hepatocyte expression of Bak1, a pro-apoptotic protein, in a model of liver injury; however, it had also an effect of preventing human gingival fibroblast apoptosis (Jung et al., 2008; Rao et al., 2018). Tacrolimus had also both pro and antiapoptotic effects in variate non-liver cellular lines (Johnson et al., 2009). In the study by Lim et al. (Lim et al., 2015), at therapeutic concentrations, none of the CNIs promoted hepatocyte cell death.

Despite the presence of multiple publications investigating functional and molecular cellular effects of MMF, cytotoxic action of MMF remains weakly defined. In a study published in 2008 (Chaigne-Delalande et al., 2008), mychophenolic acid induced a caspase dependant apoptosis in a minor cellular population. Recently, MMF therapy was associated with decreased incidence of tumors compared to untreated patients (Leckel et al., 2003). MMF treatment increased apoptosis of epithelial cells in the gastrointestinal tract, as well as apoptosis of the insular pancreatic cells, but reduced apoptosis of the renal tubular epithelium, in the glomerular and interstitial cells (Pardo-Mindan et al., 1999; Johnson et al., 2009). In contrast with other drugs, MMF therapy improved cellular viability and reduced apoptosis in primary hepatocytes, suggesting a hepatoprotector effect compared to other immunosuppressor drugs (Smiley et al., 2000). This effect was proved in our study in LT recipients with reduced fibrosis if they received MMF (Iacob et al., 2015). On the other hand, in other studies it was shown that the hepatoprotective effect of MMF is lost if this is used together with CNIs due to increased hepatocyte apoptosis. Also, based on the literature data, immunosuppression medication with serum concentrations over the therapeutic levels (>10–100 times) induces hepatocyte apoptosis. *In vitro* studies done by our team demonstrated the apoptotic and necrotic effect of the immunosuppressive medication on both cell populations hepatocytes and non-parenchymal liver cells. Studies have investigated the level of apoptosis and necrosis induced *in vitro* by different concentrations of immunosuppression medication certifying an evident relation of the toxic-necrotic and apoptotic effect with the dose of the drugs. Gene expression studies have indicated an antiapoptotic effect of MMF at doses close to the therapeutic levels through action of several apoptotic pathways (decrease of the cellular apoptosis receptors, of the genes specific to the mithocondrial apoptosis, of the effectors of apoptosis such as caspases or DFFB-CAD). In the co-culture systems, MMF + TAC association has induced antiapoptotic effects, decreasing the expression of the following genes: TRAILR1, BAK1, BAX, SMAC and of the effector proteins of apoptosis CASP8, CASP9 and CASP10.

Our results support the idea of using a low dose TAC and MMF association in case a genetic profile with a high risk of posttransplant fibrogenesis, such as the CC genotype of the TBX21 gene promoter SNP. Literature data demonstrated that SRL at therapeutic levels did not lead to the death of hepatocytes. Also, SRL proved to be less toxic compared to cyclosporine/TAC + MMF; in addition SRL + MMF led to a reduced apoptosis of hepatocytes. Other studies (Iacob et al., 2007; Manzia et al., 2011) have established also a reduced progression of posttransplant fibrosis, of the inflammation and aminotransferases levels in patients with chronic hepatitis C treated with low dose CNI and MMF.

The goal of optimum immunosuppression is to increase drug effectiveness while lowering the adverse effects, as well as a long-term graft and recipient survival with a good quality of life. Every transplant recipient needs an immunosuppression regimen tailored to their: age, comorbid conditions, transplantation indications, behavior of the allograft, complications related to immunosuppression and post-LT physiologic conditions.

This study supports the role of MMF in liver fibrosis modulation and apoptosis after LT in a clinical setting and suggests that tailoring immunosuppression could avoid fibrosis progression in the allograft.

## Data Availability

The raw data supporting the conclusions of this article can be made available by the authors upon reasonable request.
