# Supplementary material for: Host and immunosuppression-related factors influencing fibrosis occurrence post liver transplantation
Source: Front Pharmacol. 2022 Oct 18;13:1042664. doi: 10.3389/fphar.2022.1042664 (PMC9622773; doi:10.3389/fphar.2022.1042664)
Supplement: Supplementary file 1 [file Table1.DOCX]

**Table 1.** Primers used for quantification of gene expression for cellular apoptosis

| **Gene** | **NCBI Reference Sequence** | **Left primer** | **Right primer** | **Amplicon length (Bp)** |
| --- | --- | --- | --- | --- |
| CASP3 | NM_004346.4 | TTTGAGCCTGAGCAGAGACA | TTGCCTCACCACCTTTAGAA | 196 |
| CASP8 | NM_001080125.2 | TGCTTAAGGCTTTGGGAATG | TGGTAGTGTGGAATTTAGCTTTGA | 173 |
| CASP9 | NM_001229.5 | CTTACTACAGGCGTGCACCA | CTGGCCAGGTCTCTTCTCTG | 166 |
| CASP10 | NM_032977.4 | ACAGTCAGGGCCAAAAGCTA | TGAGCAAAGGAATCAGGACA | 154 |
| BAK1 | NM_001188.4 | GCCTTTGCAGTTGGACTCTC | GGGTTGGGAGCAAGTGTCTA | 167 |
| BAX | NM_001291428.2 | AGCGACTGATGTCCCTGTCT | CCTCCCAGAAAAATGCCATA | 189 |
| TNFRSF1A (TNFR1) | NM_001065.4 | CCTGGAGGACATCGAGGAG | ACCCCTCCTTTCCAGAAAAA | 150 |
| TNFRSF10A (TRAILR1) | NM_003844.4 | CATGCCAAAGGAAAATCTGG | AAACACCCTCGAAGACATGC | 164 |
| TNFRSF10B (TRAILR2) | NM_003842.5 | ATGGCTGACGCATTAAGGTT | AGTCCCGGAACAAAACACAC | 196 |
| MCL1 – isoform 1 (antiapoptotic) | NM_021960.5 | GCAGTGAGGGCTTAGGACAC | CGCCATCCTTCTAAGCAAAT | 160 |
| ASK1 (MAP3K5) | NM_005923.4 | TGGAAGAATTGGTTCGGAAA | TCACTCTCAGCCAGTCGGTA | 192 |
| RIPK1 | NM_001354930.2 | TTTCAAAGCCCACCTGAAAC | GACCATCACCACACCCTTCT | 163 |
| TNFRSF5 (CD40) | NM_001250.6 | TGTCCATCAGCAGGAGACTG | ACCCTTCCAGAACCCTTGTT | 217 |
| TNFRSF6 (FAS) | NM_000043.6 | AAAGCCAAATGAGGATTTTGAA | AGGAGGGAACCCTAAGCAAA | 198 |
| NFKB1 | NM_003998.4 | CCGTGTAAACCAAAGCCCTA | CAGCCAGTGTTGTGATTGCT | 206 |
| SMAC (DIABLO) | NM_019887.6 | CAGGGTGGAAGATTCGTGGA | TGTAGCTGAGGAGCACGAAG | 193 |
| AIFM1 | NM_004208.4 | TGGACAGTAGTTTGCCCACA | ACGACCACTTTGTCCCTGAG | 229 |
| DFFB (CAD) | NM_001282669.1 | GCCCTCAGGGTTGTGTAGTT | AGAAGGCCTGGGAAAAGTGA | 159 |
| HTRA2 | NM_013247.4 | TTGCCATCCCTTCTGATCGT | CCATGCTGAACATCGGGAAA | 178 |
